# A clinicopathological correlation of the expression of the angiopoietin-Tie-2 receptor pathway in the brain of adults with *Plasmodium falciparum* malaria

**DOI:** 10.1186/1475-2875-12-50

**Published:** 2013-02-05

**Authors:** Panote Prapansilp, Isabelle Medana, Nguyen Thi Hoan Mai, Nicholas PJ Day, Nguyen Hoan Phu, Tsin W Yeo, Tran Tinh Hien, Nicholas J White, Nicholas M Anstey, Gareth DH Turner

**Affiliations:** 1 Nuffield Department of Medicine, Centre for Tropical Medicine, Oxford University, Oxford, UK; 2 Mahidol-Oxford Research Unit, Faculty of Tropical Medicine, Mahidol University, 3rd Floor, 60th Anniversary Chalermprakiat Building, 420/6 Rajvithi Road, Bangkok, 10400, Thailand; 3 Department of Tropical Pathology, Faculty of Tropical Medicine, Mahidol University, Bangkok, Thailand; 4 Hospital for Tropical Diseases, Ho Chi Minh City, Vietnam; 5 Global Health Division, Menzies School of Health Research and Charles Darwin University, Darwin, Australia

**Keywords:** Malaria, Human, *Plasmodium falciparum*, Pathogenesis, Pathology, Cerebral, Angiopoietin, Tie-2 receptor, Immunohistochemistry, Biomarker, Blood brain barrier

## Abstract

**Background:**

Plasma angiopoietin (Ang)-2 is associated with disease severity and mortality in adults and children with falciparum malaria. However the mechanism of action of the angiopoietins in fatal malaria is unclear. This study aimed to determine whether the expression of Ang-1 and Ang-2 and their receptor Tie-2 in cerebral endothelial or parenchymal cells was specific to cerebral malaria (CM), correlated with coma or other severe clinical features, and whether plasma and CSF levels of these markers correlated with the clinical and neuropathological features of severe and fatal malaria in Vietnamese adults.

**Methods:**

Immunohistochemistry was performed for Ang-1, Ang-2 and Tie-2 on post-mortem brain tissue from fatal malaria cases and controls. Quantitative ELISA for plasma and cerebrospinal fluid levels of Ang-1, Ang-2 and Tie-2 was done to compare fatal cases with surviving patients from the same study.

**Results:**

Immunohistochemistry revealed significant differences in expression in endothelial and parenchymal cells compared to controls. However there was no significant difference in expression of these markers on endothelial cells, astroglial cells or neurons between CM and non-cerebral malaria cases. Immunostaining of Ang-1, Ang-2 and Tie-2 was also not associated with *Plasmodium falciparum*-infected erythrocyte sequestration in the brain. However Ang-1 and Ang-2 expression in neurons was significantly correlated with the incidence of microscopic haemorrhages. Plasma levels of Ang-2 and Ang-2/Ang-1 ratio were associated with the number of severe malaria complications and were significant and independent predictors of metabolic acidosis and fatal outcome.

**Conclusions:**

The independent prognostic significance of Ang-2 and the Ang-2/Ang-1 ratio in severe malaria was confirmed, although immunohistochemistry in fatal cases did not reveal increased expression on brain endothelium in cerebral *versus* non-cerebral cases. Activation of the Ang-Tie-2 pathway in severe malaria is therefore related to acidosis, number of severity criteria and outcome, but is not a specific event in the brain during cerebral malaria.

## Background

Malaria continues to be a major cause of mortality and morbidity in tropical countries [1]. Severe *Plasmodium falciparum* malaria, is characterized clinically using a series of criteria (WHO, 2000) [2], including complications such as hyperparasitaemia, metabolic acidosis, acute renal failure, severe anaemia and coma, presenting as cerebral malaria (CM). CM is a neurological syndrome comprising a potentially reversible diffuse encephalopathy, associated with retinopathy and variably with convulsions and localizing neurological signs, which are poor prognostic features [3].

The pathogenesis of CM is complex and multifactorial. A fundamental pathophysiological question in the genesis of coma in CM is how parasitized red blood cells (PRBC), remaining within the vascular space, influence parenchymal function to induce coma and death. Neurological dysfunction in severe malaria is significantly related to the sequestration of PRBC in the cerebral microvasculature, and resultant microvascular obstruction [4-7]. Both local and systemic factors may contribute to endothelial activation and blood brain barrier (BBB) dysfunction, causing secondary neuropathological processes such as neuronal and astroglial dysfunction or axonal injury [8].

The endothelium, which is the critical interface between PRBC and the brain parenchyma, is considered to be central to the pathogenesis of CM. During severe malaria infection, there is systemic activation of EC including the cerebral EC of the BBB [9,10]. Activated endothelium has increased permeability through the disruption of endothelial transmembrane proteins, and shows upregulation of a variety of surface adhesion molecules including intercellular cell adhesion molecule-1 (ICAM-1), vascular cell adhesion molecule-1 (VCAM-1), P- and E-selectin [9,11-14]. Moreover, activated ECs rapidly exocytose pre-synthesized and stored molecules from Weibel-Palade bodies (WPB) in response to changes in the vascular micro-environment. Although the main constituent of WPB is von-Willebrand factor (vWF) and its propeptide, other molecules including P-selectin, CD63, interleukin-8, endothelin-1, tissue plasminogen activator and angiopoietin (Ang)-2 are also released. The release of WPB is the initial step in the transition from a resting endothelial phenotype to activated responsive endothelium [15-18].

The Ang-Tie-2 ligand-receptor system is a key regulator of the maintenance of functional integrity in both quiescent and activated endothelium [19]. In most contexts, Ang-1-mediated Tie-2 signalling maintains endothelium in a steady state but Ang-2, which functions as an antagonist ligand of Tie-2, can destabilize this and sensitize endothelial response to inflammatory and angiogenic cytokines such as tumour necrosis factor (TNF), interleukin-1 and vascular endothelial growth factor (VEGF) [13]. In order to maintain the quiescent phenotype of endothelium, ECs need constitutive Tie-2 signalling mediated by Ang-1 binding, which is mainly released from pericytes, fibroblast and smooth muscle cells and incorporated into extracellular matrix after secretion [20]. Tie-2 phosphorylation involves several downstream signalling pathways including activating phosphatidylinositol 3-kinase and protein kinase B pathways, and inhibiting nuclear factor (NF)-κB [21]. These pathways protect cells from apoptosis and loss of intercellular junctional complexes and inhibit inflammatory responses. Exogenous stimuli such as physical damage, hypoxia or impaired endothelial nitric oxide production cause rapid exocytosis of stored Ang-2 in WPB from the ECs and competitively bind to Tie-2 receptor, which negatively interferes with constitutive Ang-1-mediated Tie-2 signalling [18,22,23]. Consequently, Ang-2-Tie-2 binding results in destabilization of endothelium and enhances EC responsiveness to other cytokines. Ang-2 can, therefore, act as an autocrine dynamic regulator of the rapid adaptive response of endothelium [13].

Recent *in vivo* studies have implicated the dysregulation of angiopoietins in the pathogenesis of severe and fatal malaria. In adults with cerebral and non-cerebral severe malaria, plasma Ang-2 has been associated with reduced endothelial nitric oxide bioavailability and mortality with Ang-2 a better predictor of death than venous lactate [24]. In children, increased plasma Ang-2 and the Ang-2/1 ratio, and reduced Ang-1 have been associated with disease severity and risk of death in CM [25-29]. Nevertheless, the mechanisms by which Ang-Tie-2 pathway contributes to CM and non-cerebral severe malaria (NCM), and fatal outcome, remain unclear.

The therapeutic use of angiopoietins has been investigated in experimental murine models of brain ischemia, where Ang-1 inhibits BBB breakdown, stimulates the recruitment of neuroblasts and promotes behavioural recovery [30]. The Ang-Tie-2 signalling pathway could potentially be a logical target for neuroprotective adjunctive therapy in CM. However their tissue specific expression and specificity in a target organ such as the brain has not been studied in human fatal malaria cases.

Therefore, in this study, the site and expression levels of the proteins Ang −1 and −2 and their receptor Tie-2 were examined in brain tissue of patients who died from malaria, to determine whether there were patterns of expression specific to cerebral malaria. Levels of the circulating forms of these proteins were examined in plasma and CSF of a subset of these patients in order to correlate circulating angiogenic protein levels and their expression patterns in the brain with the clinical manifestations of severe malaria.

## Methods

### Ethical approvals

This retrospective case–control research study used archival brain, plasma and CSF specimens of fatal and non-fatal malaria patients, who were studied in a large double-blinded trial of artemether *versus* quinine for the treatment of severe malaria in Vietnam (AQ trial) [31]. Relatives gave written informed consent for entering the study. The brain specimens of non-malaria cases (normal controls) were from a collection of routine autopsy cases at John Radcliffe Hospital, Oxford, UK, where specific written consent for retention of brain tissue for research purposes had been given. Protocols for tissue sampling, storage and use for research were approved by the Ethical and Scientific Committee of the Centre for Tropical Diseases in Ho Chi Min City, Vietnam, OXTREC 029–02, COREC (C01.002) and Human Tissue Authority license number 12217.

### Selection of cases

#### Malaria and non-malaria cases

**Figure 1:**
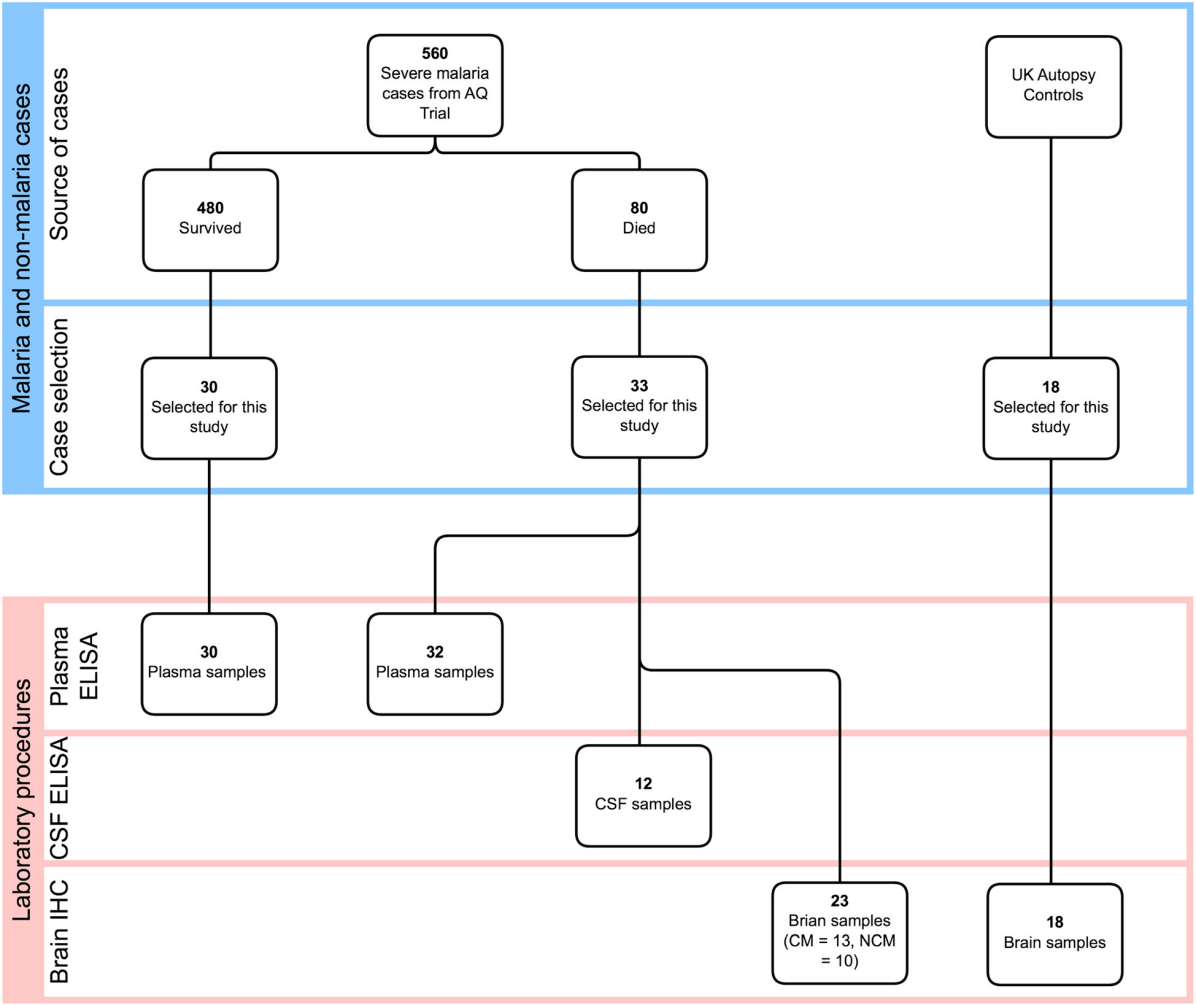
**Schematic diagram to summarise case and sample selection.** Samples were chosen through utility – cases where FFPE histological material was available, and as far as possible matching CSF and plasma samples to these cases, plus randomly selected other cases from the AQ study material to expand the numbers of fatal cases and survivors.

A total of 81 adults were included in this clinicopathological study (Figure 1). Of 81, 63 were severe malaria cases selected from the AQ trial [31], admitted at the centre for Tropical Diseases in Ho Chi Min City, Vietnam between 1991 and 1995. These were diagnosed with malaria using serial blood films and subsequent blood PCR. All were classified according to the WHO 1990 criteria for severe falciparum malaria [32]. Eighteen of the 81 were normal controls from routine autopsy at John Radcliffe Hospital (Oxford, UK). These normal controls had clinically confirmed multi-organ dysfunction and a variety of different causes of death, with normal appearances on subsequent neuropathological examination as previously detailed [33].

### Brain specimens

In total, 41 cases were used. Twenty-three fatal malaria patients from the AQ study were examined, 13 of which were defined on WHO criteria as dying with CM and 10 were non-cerebral malaria (NCM). Brain sections from 18 normal controls from the UK were also used. For the malaria patients, brain specimens were collected at autopsy within 24 hours (median 7 hours), fixed in 10% formalin, then embedded in paraffin and processed using standard methods as previously described [34]. Clinical details of fatal malaria and control cases are shown in Additional files 1 and 2, respectively.

### Plasma and CSF specimens

Plasma samples from 62 out of 63 severe malaria cases were used for the quantitation of the angiogenic proteins by ELISA. CSF samples were available from only 12 severe malaria cases. Venous blood samples were taken immediately after admission into EDTA anticoagulant. Plasma samples were generated by centrifugation at 5,000 *g* for 3 min, then aliquoted and stored at −80°C until use. CSF was taken when the procedure was clinically indicated, aliquoted neat without any additives, spun and frozen at −80°C until use [35].

### Clinical, biochemical and neuropathological data for correlations

The cases used in this retrospective study had subsequent extensive clinical, biochemical and neuropathological investigations [31,33-38]. These existing data were correlated with immunostaining scores and plasma levels of Ang-1, Ang-2 and Tie-2 proteins generated from this study. These included assessment of the number of criteria of severe malarial disease, coma status, biochemical evidence of other end organs disease such as creatinine level for renal failure, liver function tests and lactate, peripheral blood counts for anaemia, white cell count and platelet levels, and neuropathological quantitation of sequestration, malarial haemorrhages and degree of oedema in individual cases. Data on CSF levels of the neurotransmitters quinolinic acid and kynurenine ratios [35] were also used.

### Detection and quantification of Ang-1, -2 and Tie-2 proteins in the brain

#### Immunohistochemistry

Immunohistochemistry (IHC) was performed for three different markers of Ang-Tie-2 pathway including the Ang-1 protein, Ang-2 and Tie-2 receptor. Staining was performed on 4 mm sections of formalin-fixed paraffin embedded brain blocks cut onto coated immunoslides (SupaFrost), from all malaria and control cases. The sections were dewaxed in xylene, rehydrated in graded alcohol series and then underwent microwave antigen retrieval in Tris-EDTA pH 8 for 10 min. The primary antibodies used were: Ang-1 (mouse polyclonal, A0604, Sigma, UK, 1:20 dilution), Ang-2 (rabbit polyclonal, Ab65835-100, Abcam, UK, 1:50 dilution) and Tie-2 (rabbit polyclonal, SC-9026, Santa Cruz, USA, 1:100 dilution). Detection of bound antibody was visualized using the high-sensitivity Novolink™ Polymer detection system (Leica Biosystems, UK). Negative controls comprised sections immunostained as above with omission of the primary antibody.

Three areas of the brain from each case were examined including cortex, diencephalon and brain stem, to determine any site specific variations in the patterns of immunoreactivity. With each brain area, the pattern and intensity of staining were examined in three different cellular components including vessels, neurons and astroglial cells.

#### Quantitation of vascular immunostaining

Vessels were considered positive for Ang-1, Ang-2 and Tie-2 when any parts forming the vessel including ECs, basement membrane, pericytes or smooth muscles were stained. The number of positive vessels in 150–300 fields in each slide at x200 magnification was used to calculate the number of positive vessels per centimetre squared. The continuous variable of number of positive vessels per cm^2^ was converted to a semiquantitative Vascular Expression Score (VasExp) as follows: Ang-1; low = 0–20 vessels/cm^2^, moderate = 20–40 vessels/cm^2^, high ≥40 vessels/cm^2^: Ang-2; low = 0–200 vessels/cm^2^, moderate = 200–400 vessels/cm^2^, high ≥400 vessels/cm^2^: Tie-2; low = 0–10 vessels/cm^2^, moderate = 10–20 vessels/cm^2^, high ≥20 vessels/cm^2^. Both scores were used in histopathological and clinicopathological correlations where continuous or semiquantitative categorical measurements where most appropriate.

#### Semiquantitation of neuronal and astroglial immunostaining

Ang-1 and Ang-2 expression on neurons and astroglial cells was classified in a semi-quantitative scoring system of both the number of cells and the intensity of expression. Cell numbers were classified according to a percentage score as follows: score 0 = percentage of stained cells 1% or less, score 1 = 1-10%, score 2 = 10-50% score 3 ≥50%. The intensity score was defined as: score 0 = no staining, score 1 = weak staining (+/−), score 2 = moderate staining (+), score 3 = strong staining (++). The neuronal expression (NeuExp) of Ang-1 and Ang-2 was represented as the sum of the percentage score and intensity score (NeuScore) and also divided into two groups of “low” (low NeuExp, NeuScore ≤3) and “high” neuronal expression (high NeuExp, NeuScore ≥4). Astroglial expression (GliExp) of Ang-1 and Ang-2 was classified based on a similar system combining the percentage score with the intensity score into “low” (low GliExp, GliScore ≤3) and “high” astroglial expression (high GliExp, GliScore ≥4). However, due to much lower expression of Tie-2 on neurons and astroglia cells, it could not be quantitated using the same scoring system as Ang-1 and Ang-2. Rather than using both the number of cells and the intensity of staining, the semi-quantitative scoring system for Tie-2 expression on neurons and astroglial cells was based on the number of cells staining as follows: “low” neuronal or astroglial expression (low NeuExp or GliExp) = percentage of stained cells 1% or less and “high” neuronal or astroglial expression (high NeuExp or GliExp) = percentage of stained cells >1%.

#### Quantification of Ang-1, -2 and Tie-2 proteins in the plasma and CSF

Plasma and CSF concentrations of Ang-1, Ang-2 and Tie-2 were measured by quantitative ELISA (R&D Systems, UK), according to the manufacturer’s instructions, each sample tested in duplicate. Concentrations were interpolated from 5-parameter-logistic (5-PL) standard curve fitting, using the manufacturer’s recombinant human Ang-1, Ang-2 and Tie-2 proteins.

A total of 62 plasma samples and 12 CSF samples from patients of severe malaria were examined, all in duplicate. Twenty-two of the 62 plasma samples and 12 CSF samples were from fatal malaria cases included in the immunohistochemical analysis. The other 40 plasma samples were from severe malaria patients in the same AQ trial, selected randomly, including fatal cases (n = 10/40) and survivors (n = 30/40).

#### Statistical methods

Analyses were performed using Stata/SE 9.2 (StataCorp LP, Texas, USA), with a *P* value less than 0.05 taken as being statistically significant. Continuous variables were tested for normal distribution with the Kolmogorov-Smirnov test, (this showed that the distribution of all continuous variables measured was not normal). Thus data were summarized with median and interquartile values, and non-parametric tests, including Kruskal-Wallis, Mann–Whitney U test, and Wilcoxon matched-paired signed-ranks test were used to compare groups where appropriate. Correlations between continuous variables were tested by Spearman’s rank correlation.

For IHC expression score data: due to the correlated nature of data obtained from different areas of the brain within the same patient (three areas of the brain from one patient), intraclass correlation, which affected the standard errors of the estimates, were taken into account by using clustered robust standard errors (one patient as one cluster) with univariate logistic or ordered logistic regressions where appropriate.

The prognostic value of plasma angiopoietins, Tie-2 and other biomarkers was assessed by calculating the area under the receiver operating characteristic curve (AUROC). The AUROCs were compared nonparametrically by DeLong’s method.

Multivariate analyses were conducted by either linear regression or logistic regression, depending on the nature of the dependent variable. Stepwise techniques were used manually in the light of clinical judgments where appropriate.

## Results

### Patterns of Ang-1, Ang-2 and Tie-2 immunostaining in the brain

**Figure 2:**
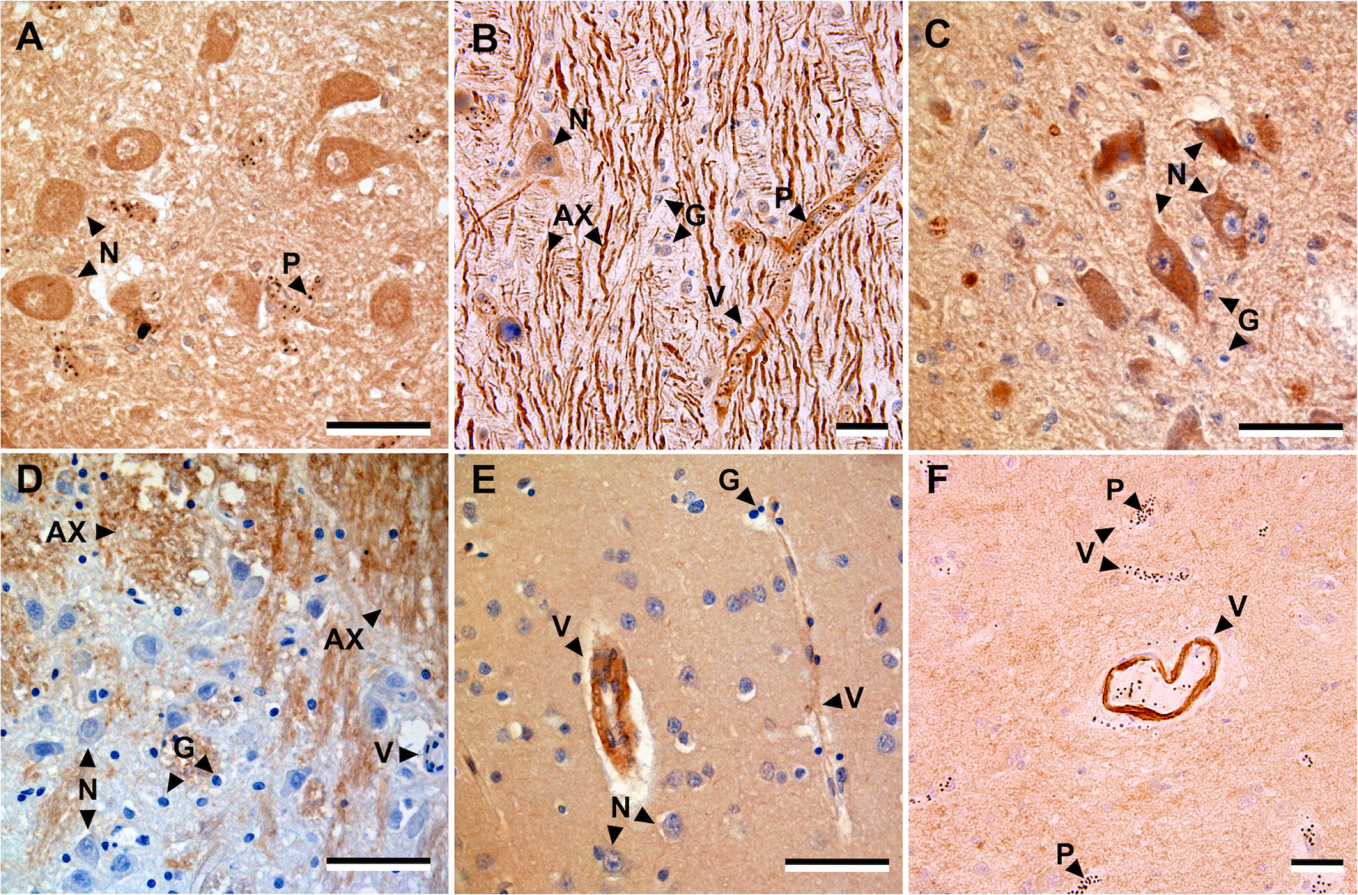
**The immunostaining of Ang-1, Ang-2 and Tie-2 in brain sections from fatal malaria cases (A, B, C, F) and fatal non-malaria cases (D, E).** There was heterogeneity in expression patterns of Ang-1, Ang-2 and Tie-2 between patients and within different brain regions in each patient. (**A, D**) Ang-1 was more commonly seen on larger neurons or astroglial cells, perivascular smooth muscles and pericytes, but not usually on endothelial cells (ECs). Strong staining was occasionally observed on neuropil and axonal bundles. (**B, E**) Strong Ang-2 labelling on axonal bundles, large neurons, astroglial cells, perivascular smooth muscles, pericytes, ECs and some parasitized red blood cells. (**C, F**) Strong Tie-2 staining of large and small neurons, astroglial cells, perivascular smooth muscles, pericytes and ECs. (**F**) A larger vessel showing Tie-2 staining whilst surrounding capillaries, showing significant parasitized erythrocyte sequestration, are completely negative. All sections were counterstained with haematoxylin. Scale bars = 60 μm. Abbreviations: AX = axons, G = astroglial cells, N = neurons, P = parasitized red blood cells, V = vessels.

Ang-1, Ang-2 and Tie-2 staining could be found in the cytoplasm and/or nucleus of a range of cells, including neurons, astroglial cells, ECs, pericytes, and perivascular smooth muscle cells, in the brain sections of malaria and non-malaria patients (Figure 2). There was considerable heterogeneity in the expression of Ang-1, Ang-2 and Tie-2 between patients and different brain areas of each patient. The expression of Ang-1 and Ang-2 was more commonly seen on larger neurons or astroglial cells than smaller neuron or astroglial cells. All three proteins could be seen staining vascular components including ECs, pericytes and perivascular smooth muscles, but Ang-1 expression was almost never seen on ECs. All three angiogenic markers were also observed in axonal fibres, intravascular serum and red blood cells either with or without parasites. However, Tie-2 staining on astroglial cells was exclusively found in malaria cases and not constitutive in controls.

### Comparative quantitation of Ang-1, Ang-2 and Tie-2 expression between different areas of the brain

The expression of each marker was compared between the three different brain areas and in different cellular components. Detailed analysis of staining patterns of all cases (pooled malaria cases and non-malaria controls) and of malaria cases only is presented in Additional files 3 and 4, respectively.

### Immunostaining of Ang-1, Ang-2 and Tie-2 differentiates malaria cases from controls but not cerebral malaria from non-cerebral malaria

**Figure 3:**
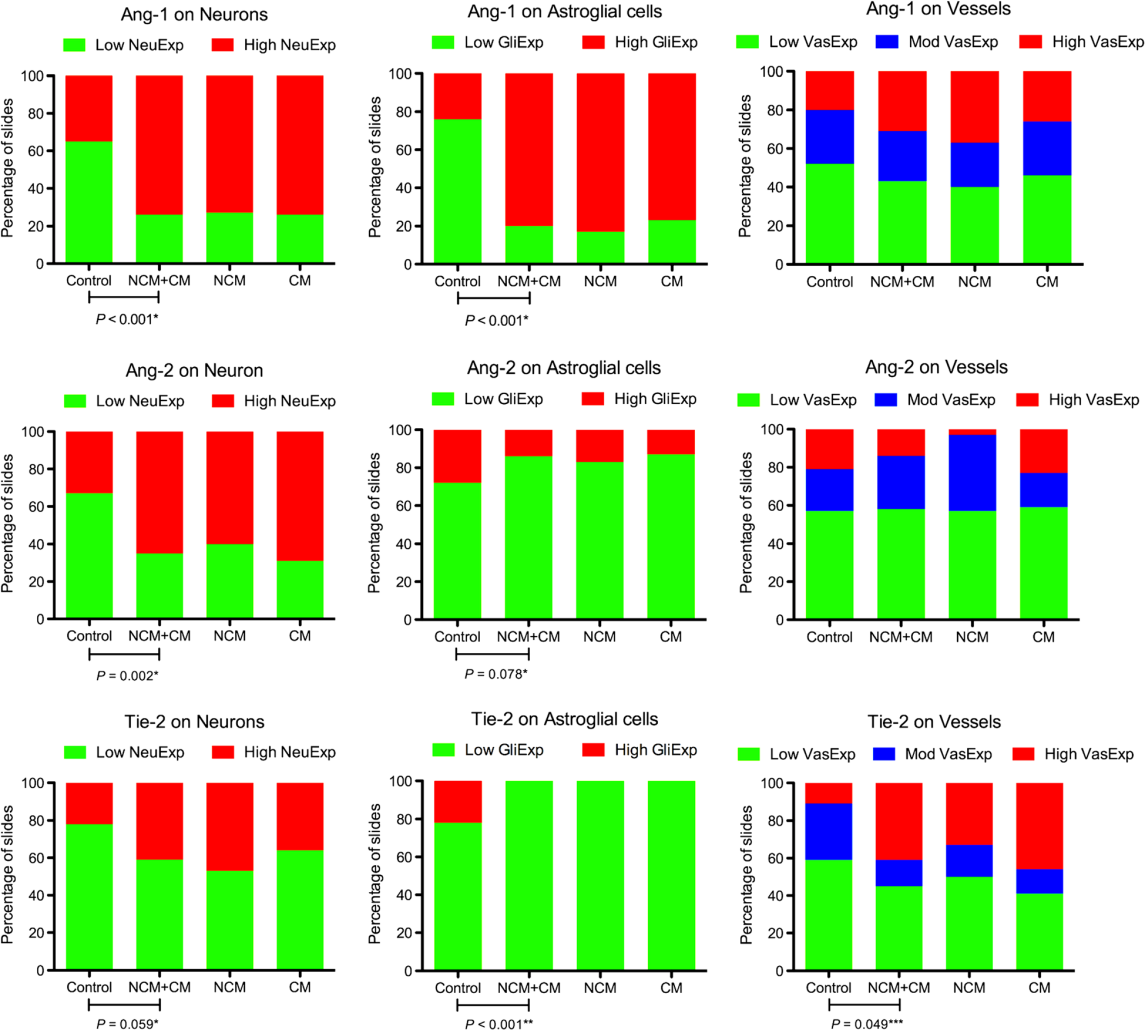
**Immunostaining patterns for Ang-1, Ang-2 and Tie-2 differentiated control from malaria cases.** Graphs show percentages of cases positive according to marker, cell components and expression scores. The immunostaining patterns of Ang-1, Ang-2 and Tie-2 in malaria cases were significantly different from those in non-malaria cases, especially the staining for neuronal and astroglial components. However, these proteins were not differentially expressed in CM compared to NCM. (*Logistic Regression with clustered robust standard errors, **Fisher's Exact Test, ***Ordered Logistic Regression with clustered robust standard errors).

**Figure 4:**
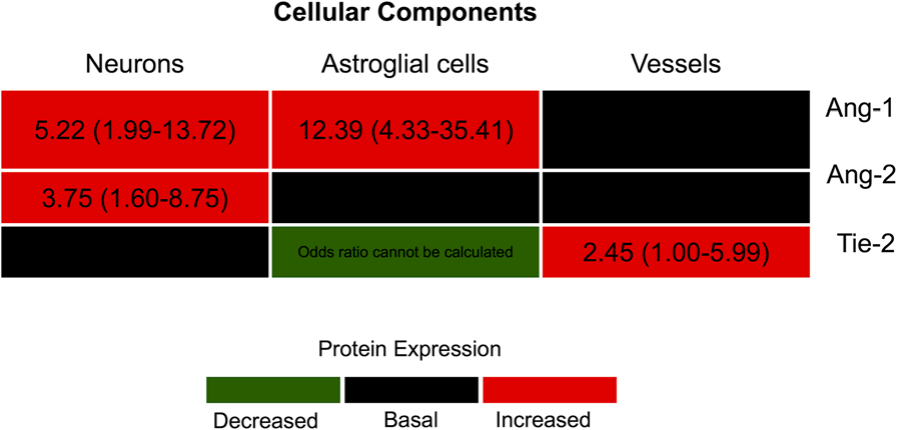
**Summary of the expression pattern of angiogenic proteins in the brain of fatal malaria patients compared with fatal non-malaria patients.** Using immunohistochemistry, increased expression of Ang-1 in neurons and astroglial cells, increased expression of Ang-2 in neurons, and increased expression Tie-2 in vessels were observed in the brain of fatal malaria patients compared to fatal non-malaria patients. Decreased expression of Tie-2 in astroglial cells was observed in fatal malaria cases. Values are odds ratios (brackets = 95% confidence intervals) of higher expression scores (NeuExp, GliExp or VasExp) in malaria compared to control patients.

Because the circulating levels of Ang-1, Ang-2 and Ang-2/Ang-1 ratio have been reported to be clinically informative biomarkers for CM, we examined the expression patterns of proteins Ang-1, Ang-2 and their receptor Tie-2 in the brain tissue of patients dying of CM and compared them to NCM. No significant differences were found between the immunostaining patterns of Ang-1, Ang-2 and Tie-2 on all three cellular components including neurons, astroglial cells and vessels in CM cases compared to NCM (Figure 3). However, there were significant differences in the patterns of immunostaining of Ang-1, Ang-2 and Tie-2 in malaria cases compared to non-malaria cases, especially in neurons and astroglial cells. Summaries of the staining patterns and odds ratios are shown in Figure 4.

### Ang-1

The incidence of high Ang-1 expression on neurons and astroglial cells was greater in malaria cases (73.91%, 79.71%, respectively), compared to non-malaria cases (35.19%, 24.07%, respectively) (*P <* 0.001). Ang-1 expression on vessels did not differ between malaria and non-malaria cases.

### Ang-2

The incidence of high Ang-2 expression on neurons was greater in brain sections of malaria cases (65.22%), compared to non-malaria cases (33.33%) (*P =* 0.002). Ang-2 astroglial expression was lower in malaria compared to non-malaria (*P =* 0.078); which became statistically significant after adjusting for age and sex (*P =* 0.038).

### Tie-2

Reduced expression of Tie-2 was observed in astroglial cells of malaria cases, all slides of malaria cases (100%) did not express Tie-2 on astroglial cells, compared to 77.78% of non-malaria cases (*P <*0.001*,* Fisher’s exact). The vascular expression of Tie-2 was significantly higher in malaria cases (high VasExp 40.58%), compared to non-malaria cases (low VasExp 11.1%) (*P =* 0.049). There was a trend of increased incidence of Tie-2 neuronal expression in malaria compared to non-malaria (*P =* 0.059).

### Increased expression of Ang-1 and Ang-2 in neurons is associated with microscopic haemorrhages

**Figure 5:**
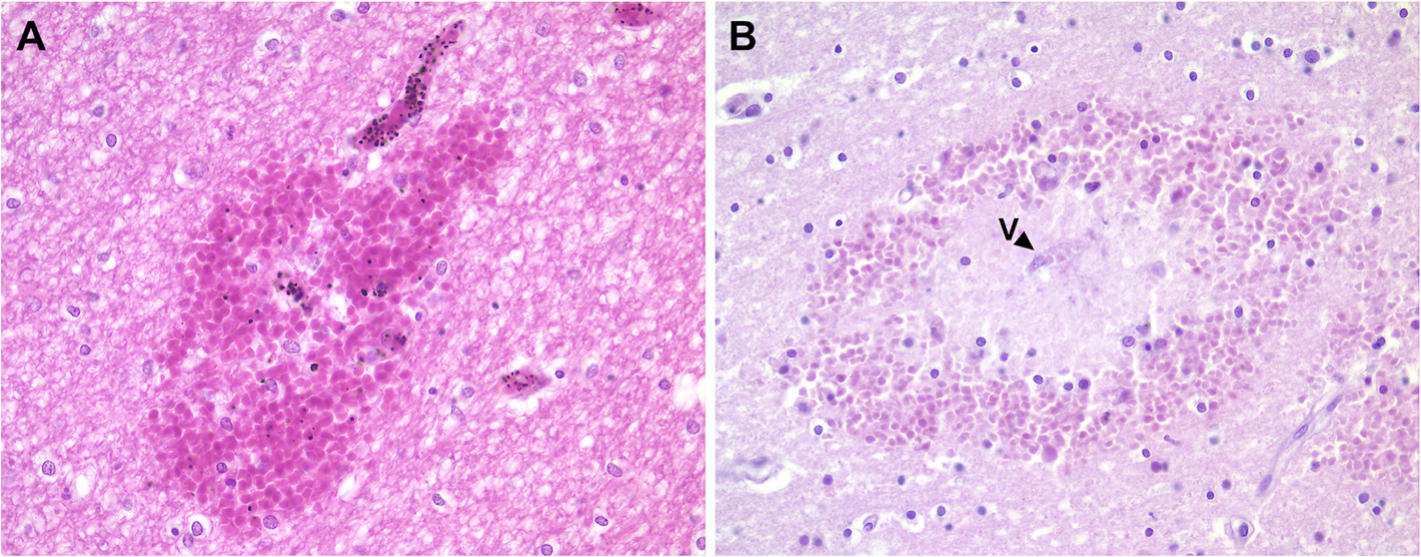
**Malaria hemorrhages.** Photomicrographs to demonstrate the histological characteristics of simple petechial haemorrhage (**A**) and a classical ring haemorrhage (**B**) in the brain tissues of a case of cerebral malaria. The simple petechial haemorrhage shows a feeding vessel containing sequestered parasitized erythrocytes and an area of leakage of infected and uninfected erythrocytes into the brain parenchyma. In contrast the ring haemorrhage shows a central vessel (V), surrounded by a zone of clearing and astroglial response, with an outer zone of erythrocytes and leukocytes which have been extravasated from the ruptured vessel. Such haemorrhages may contain fibrin platelet thrombi in the vascular lumen (Haematoxylin and Eosin staining x400).

Microscopic cerebral haemorrhages are a common but non-specific neuropathological finding in CM [8]. Two main types of haemorrhage are seen including simple petechial haemorrhage or organized ring haemorrhages (Figure 5). Both types of haemorrhage are not exclusively specific to CM, but also present in NCM and other diseases such as bacterial infections and thrombotic phenomena such as bone marrow embolism or barotrauma. They represent focal damage to the microvasculature and hence the regulation of EC barrier function. Therefore, we examined the correlation between immunostaining patterns of Ang-1, Ang-2 and Tie2 and microscopic haemorrhage in the brain.

Within the brain sections of malaria cases in this study, 40.6% showed haemorrhages. Petechial haemorrhages (88.9%) were more common than ring haemorrhage (11.1%). No significant difference in the incidence of haemorrhages was found between CM and NCM in this series. There was no correlation between the vascular or astroglial expression of Ang-1, Ang-2 or Tie-2 and the incidence or number of haemorrhages per cm^2^. However, high NeuExp of Ang-1 (OR = 7.11, 95%CI = 1.50-33.64) was associated with the presence of microscopic haemorrhages (*P =* 0.013).

Taking all clinical groups (malaria and non-malaria) into account, high NeuExp of Ang-1 (OR = 13.30, 95%CI = 3.27-54.09) and Ang-2 (OR = 5.5, 95%CI = 1.77-17.01) was significantly associated with the presence of haemorrhage (*P =* 0.003, *P <*0.001, respectively).

### Immunostaining of Ang-1, Ang-2 and Tie-2 is not associated with parasite sequestration in the brain

No correlation was found between the degree of PRBC sequestration in cerebral microvessels (measured as the percentage of vessels containing histologically observed PRBC) and the expression of any of the angiogenic proteins in either vessels, astroglial cells or neurones.

### CSF levels of circulating Ang-1, Ang-2 and Tie-2 are much lower than those in plasma

Sixty-two plasma samples of malaria patients were included. Only a limited number of CSF samples were available from cases where IHC had been performed on the corresponding case (n = 12). Although this did not provide enough power to determine the association of the CSF levels of the angiogenic markers with the clinical groups or parameters, the CSF levels of Ang-1, Ang-2 and Tie-2 had not been previously examined in malaria patients. Median and inter-quartile range of CSF and plasma levels of Ang-1, Ang-2 and Tie-2 are shown in Table 1. There were no correlations between baseline plasma concentrations of Ang-1, Ang-2 and Tie-2. The CSF concentrations of all three markers were significantly lower than the plasma concentration of themselves in the same cases (ranging from three to 800 times) (*P* = 0.0005, Wilcoxon matched-paired signed-ranks test). However this group was too small to determine any association between CSF levels and either plasma levels of the same marker, or clinical outcome.

**Table 1:** Correlation of circulating Ang-Tie-2 concentrations and clinical complications in malaria patients

		**Ang-1 (pg/ml)**	**Ang-2 (pg/ml)**	**Tie-2 (pg/ml)**	**Ang-2/Ang1 Ratio**
**CSF (n=12)**		10.27 (8.1-11.1)	609 (388–1059)	247.6 (119–343)	-
**Plasma (n=62)**		543.15 (401–837)	21126 (11590–33595)	28232 (22425–33418)	39.25 (23.14-62.56)
**Fatal outcome**	**No (n=30)**	591.36 (377.8-862.7)	14883 (9369–24849)	28232 (17288–33106)	30.55 (18.00-48.28)
	**Yes (n=32)**	495.33 (401.0-825.2)	28215 (17040–42921)	28213 (22684–34395)	42.54 (30.57-70.07)
	P-Value*	0.95	**0.002**	0.68	**0.029**
**Hyper-parasitaemia**	**No (n=41)**	688.94 (412–976)	18706 (11590–33565)	26013 (22684–34277)	31.26 (18.39-53.22)
	**Yes (n=21)**	495.33 (377–615)	23863 (16110–34426)	28709 (22425–32990)	55.63 (41.60-69.26)
	P-Value*	0.08	0.36	0.88	**0.01**
**Jaundice**	**No (n=19)**	459.77 (263–787)	9692 (7064–12949)	22907 (15334–29245)	21.94 (8.18-60.95)
	**Yes (n=43)**	591.36 (401–963)	25041 (18288–41865)	28824 (22981–37659)	41.60 (30.17-63.16)
	P-Value*	0.13	**<0.0001**	**0.016**	**0.031**
**Anaemia**	**No (n=23)**	495.33 (401–787)	12848 (9369–24519)	29245 (24960–33418)	28.10 (18.00-44.05)
	**Yes (n=39)**	555.18 (377–988)	26565 (13657–41865)	23800 (19090–35373)	41.70 (30.55-71.30)
	P-Value*	0.92	**0.004**	0.19	**0.009**
**Acute renal failure**	**No (n=23)**	495.33 (377–713)	11615 (8376–18706)	27908 (22684–32990)	30.10 (18.00-43.75)
	**Yes (n=39)**	664.38 (401–988)	26788 (18288–43978)	28709 (21318–37659)	41.60 (30.17-69.26)
	P-Value*	0.06	**<0.0001**	0.58	0.06
**Hypoglycaemia**	**No (n=44)**	567.21 (401–812)	18497 (9829–26217)	28805 (22907–34825)	31.60 (18.78-48.28)
	**Yes (n=18)**	519.24 (377–963)	32948 (13657–45867)	23241 (19853–32679)	57.99 (37.34-85.16)
	P-Value*	0.98	**0.007**	0.29	**0.019**
**Shocked**	**No (n=38)**	173.50 (401–988)	19932 (11590–31282)	28767 (22684–37659)	33.94 (18.78-56-24)
	**Yes (n=24)**	459.77 (389–591)	24191 (11841–34918)	27699 (21817–31751)	43.62 (29.77-76.06)
	P-Value*	0.053	0.36	0.48	0.13
**Hyperlactataemia**	**No (n=39)**	688.94 (401–988)	18706 (10415–33565)	27718 (21171–37659)	32.66 (22.77-59.84)
	**Yes (n=23)**	495.33 (377–713)	24601 (12444–35352)	28786 (23501–32990)	43.48 (23.14-70.88)
	P-Value*	0.14	0.36	0.69	0.2
**Cerebral malaria**	**No (n=29)**	495.33 (308–812)	21540 (13657–31021)	24960 (19090–37818)	41.60 (29.66-62.56)
	**Yes (n=33)**	579.28 (436–850)	18706 (9692–34426)	28709 (22918–32990)	37.55 (20.65-63.61)
	P-Value*	0.41	0.75	0.49	0.59
**Pure cerebral malaria**	**No (n=54)**	519.24 (377–837)	24191 (12848–34485)	28633 (22425–35373)	41.35 (29.66-66.28)
	**Yes (n=8)**	713.50 (471–862)	7237 (5178–11321)	25407 (19415–29744)	8.18 (5.65-20.55)
	**P-Value**	0.31	**<0.0001**	0.27	**0.002**

Values are presented as median (IQR), *Mann–Whitney U test.

### Plasma levels of Ang-2 and Ang-2/Ang-1 ratio are significant predictors of fatal outcome

The level of Ang-2 and the ratio of Ang-2/Ang-1 (plasma samples collected at time of admission) were significantly higher in patients with fatal outcome than survivors (*P =* 0.002 and *P =* 0.029, respectively). Ang-1 and Tie-2 concentrations were not associated with fatal outcome (Table 1).

In order to assess the prognostic value of Ang-2 and Ang-2/Ang-1 ratio in assessing a fatal outcome in severe malaria patients, receiver operating characteristic (ROC) curves were plotted, and the areas under the ROC (AUROC) were compared. ROC curves of other laboratory measures previously shown to be important prognostic markers of death in severe malaria were also plotted and compared with those of Ang-2 and Ang-2/Ang-1 ratio. These included plasma creatinine, lactate and TNF. Ang-2 has an area under curve (AUROC) of 0.727 (95%CI = 0.6-0.85), and Ang-2/Ang-1 ratio has an AUROC of 0.662 (0.522-0.802). The prognostic values of Ang-2 and Ang-2/Ang-1 ratio were not significantly different from each other or those of plasma creatinine, lactate and TNF (Table 2). These results confirm the utility of Ang-2 and Ang-2/Ang-1 ratio as predictive markers of fatal outcome in severe malaria as shown in previous studies [24,39].

**Table 2:** Comparison of prognostic value of plasma marker concentrations for fatal outcome

**Marker**	**AUC (95%CI)**	**Compared to Ang-2**	**Compared to Ang-2/Ang-1 Ratio**
Ang2	0.727 (0.600-0.854)		
Ang2/Ang1 Ratio	0.662 (0.522-0.802)	0.82, 0.36	
Creatinine	0.708 (0.575-0.840)	0.09, 0.77	0.22, 0.64
Lactate	0.666 (0.528-0.804)	0.4, 0.52	0.01, 0.93
TNF (n=32)	0.696 (0.528-0.863)	0, 1	0.57, 0.75

AUC = area under ROC curve, Data are *x*^2^, *P-*value unless otherwise indicated. All *X*^2^ and *P*-value are calculated from pairwise nonparametric comparison of areas under ROC curve.

A multivariable logistic regression model was constructed to assess whether these angiogenic proteins were independently associated with death, using fatal outcome as the dependent variable and the angiogenic proteins as well as creatinine, lactate and TNF as the independent variables. In this model, none of the markers were independently significantly associated with death. Then backward stepwise approach was used and showed that Ang-2 (OR = 1.000036 per pg/ml, 95%CI = 1.000004–1.000068) and lactate (OR = 1.21 per mmol/L, 95%CI = 1.032–1.435) were independently associated with fatal outcome. Ang-2 and lactate remain significant prognostic markers after adding into the model other clinical and biochemical parameters, including age, sex, presence of shock, presence of hypoglycaemia, admission Glasgow coma score (GCS), admission haematocrit and parasite count.

### Plasma levels of Ang-2 and Ang-2/Ang-1 ratio are associated with multiple clinical complications of severe malaria

High plasma levels of Ang-2 were significantly associated with the presences of several complications of severe malaria including fatal outcome, jaundice, anaemia, acute renal failure and hypoglycaemia (Table 1). High Ang-2/Ang-1 ratios were also associated with the same clinical complications as Ang-2, but with slightly lower magnitude. Hyperparasitaemia was associated with high Ang-2/Ang-1 ratio and low Ang-1 but not independently associated with raised Ang-2. High plasma levels of Tie-2 were associated with the presence of jaundice but not the other complications.

### Plasma levels of Ang-2 is not a biomarker for CM but a prognostic marker for progressive multi-organ dysfunction in severe malaria

The presence of cerebral malaria was not related to the plasma concentrations of Ang-1, Ang-2 and Tie-2; however, patients with “pure” cerebral malaria (cerebral malaria patients without the other complications of severe malaria according to WHO 2000 criteria) had significantly lower Ang-2 concentration and Ang-2/Ang-1 ratio than the other patients (Table 1).

Plasma Ang-2 concentrations were significantly associated with the number of severe complications of severe malaria occurring in a patient (*P <*0.0001, Kruskal-Wallis) (Table 3). This indicated that Ang-2 could be a prognostic marker of progressive multiple organ dysfunction in severe malaria. Conversely, there were no correlations between Ang-1 or Tie-2 concentrations and the number of severity criteria.

**Table 3:** Baseline Ang-2 plasma concentration stratified by the number of WHO 2000 clinical criteria for severe malaria

**No. of severe criteria**	**N**	**Ang-2 (pg/ml)**	**Ang-2/Ang1**
1	16 (25.8%)	9730 (7237–15240)	20.55 (8.18-55.63)
2	28 (45.2%)	22616 (14883–34889)	38.53 (28.74-61.15)
3	8 (12.9%)	23532 (15977–28215)	51.66 (17.19-67.77)
4	9 (14.5%)	34485 (31282–43978)	41.60 (37.34-70.88)
5	1 (1.6%)	58192	94
Kruskal-Wallis		P <0.0001	P = 0.10

### Correlation of clinical and laboratory parameters with plasma levels of Ang-1, Ang-2 and Tie-2 proteins

Spearman’s rank correlations were calculated between plasma concentrations of Ang-1, Ang-2 or Tie-2 and a number of clinical and biochemical markers in malaria patients at admission, including GCS, respiratory rate, haematocrit, white blood cell count, platelets, parasite count, liver function tests, creatinine, glucose, electrolytes, lactate, TNF, and arterial blood gas measures. The summary of correlations of some important markers of malaria severity was presented in Table 4.

**Table 4:** Summary of correlations between plasma angiogenic markers and laboratory parameters

		**Ang-1**	**Ang-2**	**Tie-2**	**Ang-2/Ang1 Ratio**
**Body temperature**	rho, P-value*	0.2788, 0.029	ns	0.441, <0.0001	
	**n**	61		62	
**Haematocrit**	rho, P-value*	ns	−0.368, 0.003	ns	ns
	**n**		62		
**Parasite count**	rho, P-value*	ns	ns	ns	0.3, 0.018
	**n**				61
**Platelets**	rho, P-value*	0.305, 0.018	−0.294, 0.022	ns	−0.442, <0.0001
	**n**		60		59
**White blood cells**	rho, P-value*	ns	0.471, <0.0001	ns	ns
	**n**		59		
**BUN**	rho, P-value*	ns	0.733, <0.0001	ns	0.554, 0.002
	**n**		27		27
**Creatinine**	rho, P-value*	ns	0.619, <0.0001	ns	0.293, 0.02
	**n**		62		61
**Potassium (K)**	rho, P-value*	ns	0.311, 0.027	ns	ns
	**n**		50		
**SGOT**	rho, P-value*	0.317, 0.014	0.415, 0.001	ns	ns
	**n**	59	60		
**SGPT**	rho, P-value*	ns	0.283, 0.028	ns	ns
	**n**		50		
**TNF**	rho, P-value*	0.416, 0.017	ns	ns	ns
	**n**	32			
**Lactate**	rho, P-value*	ns	ns	ns	ns
	**n**				
**Pyruvate**	rho, P-value*	ns	ns	ns	ns
	**n**				
**PH**	rho, P-value*	ns	−0.42, 0.004	ns	ns
	**n**		44		
**pCO2**	rho, P-value*	ns	−0.4832, <0.0001	ns	−0.438, 0.003
	**n**		44		43
**CSF Kynurenine**	rho, P-value*	ns	ns	ns	ns
	**n**				
**CSF Quinolinic Acid**	rho, P-value*	ns	0.711, <0.0001	ns	0.625, <0.0001
	**n**		31		30
**CSF Picolinic Acid**	rho, P-value*	ns	0.753, <0.0001	ns	0.708, <0.0001
	**n**		31		

*Spearman's rank correlation. No adjustments for multiple comparison have been made. All parameters were measured from plasma samples (except indicated as CSF) at time of admission. ns = not significant.

Plasma Ang-1 concentration was positively correlated with the admission levels of platelets. This finding may reflect platelet production and release of circulating Ang-1 [40]. However Ang-2 and Ang-2/Ang-1 ratio were negatively correlated with platelet counts. There were positive correlations between body temperature and concentrations of Ang-1 and Tie-2, raising the possibility that these proteins might play a role in body temperature regulation in response to the disease. Plasma Ang-2 was correlated with several indicators of disease activity/severity, including increased white cell count, increased liver enzymes (SGOT and SGPT), decreased renal function (elevated BUN, creatinine and potassium) and metabolic acidosis (low pH, high pCO_2_). Moreover, plasma Ang-2 concentration was strongly positively correlated with CSF concentrations of quinolinic acid and picolinic acid, which have been previously shown to be strongly associated with acute renal failure in this group [38]. There were no correlations between admission parasite count and the plasma levels of Ang-1 Ang-2 or Tie-2, but admission parasite count was significantly correlated with the ratio of Ang-2/Ang-1.

Interestingly lactate and pyruvate levels, that could also contribute to metabolic acidosis, were not correlated with Ang-1, Ang-2 and Tie-2 levels. This finding suggested that Ang-2 was correlated with metabolic acidosis in a manner independent of lactate levels (Spearman’s rho = 0.09, *P =* 0.47).

### Plasma levels of Ang-2 and lactate were independently associated with metabolic acidosis

As described above, plasma Ang-2 levels were closely correlated with several established prognostic indices of malaria disease severity and several factors indicating disease activity. As the various clinical manifestations and laboratory parameters of severe malaria are interlinked, a backward stepwise multivariable linear regression model was constructed to determine the factors contributing to plasma Ang-2 level, with plasma level of Ang-2 as the dependent variable and haematocrit, parasite count, platelets, white cell counts, creatinine, SGOT, and lactate as possible explanatory factors. All of these parameters independently and significantly associated with the level of Ang-2 in this model and together they are accounted for 67% (R^2^ = 0.677) of the variance in Ang-2 (Model A, Table 5).

**Table 5:** Summary of multivariate regression models

**Model**	**Dependent variable**	**Regression type**	**Independent variables**	**Coefficients (95%CI)**	**Standardized Coefficients**	***P***
A	Plasma Ang-2	Linear regression	Haematocrit	−506.65 (−933 to −80)	−0.2294042	0.021
			Parasite count	0.0116 (0.004 to 0.019)	0.2893166	0.003
		R^2^=0.677	Platelet	−0.200 (0.333 to −0.067)	−0.27261	0.004
			White cell	1.260 (0.746 to 1.774)	0.4790173	<0.0001
			Creatinine	1965 (20.2 to 3911)	0.2087163	0.048
			SGOT	44.60 (19.61-69.58)	0.3070279	0.001
			Lactate	−1539 (−2416 to −661.7)	−0.3230233	0.001
			Constant	23369 (3808 to 42930)	-	0.02
B	Plasma Ang-2	Linear regression	Haematocrit	−591.39 (−1111 to −71)	−0.275	0.027
			Parasite count	0.0111 (0.002 to 0.020)	0.280	0.017
		R^2^=0.623	White cell	1.44 (0.832 to 2.057)	0.352	<0.0001
			SGOT	71.96 (23.07 to 120.85)	0.530	0.005
			Constant	9200 (−12672 to 31072)	-	0.399

There was no correlation between lactate and Ang-2 in the univariate analysis, and that the multivariable logistic regression model predicting death as an outcome showed that Ang-2 and lactate were independent factors contributing to death; however, in this linear regression model (Model A, Table 5), lactate was a significant independent contributing factor to Ang-2. This suggested that there were other confounding factors related to the levels of Ang-2 and lactate. This could be metabolic acidosis, which is closely related as a complication of severe malaria to renal failure and hyperlactatemia, as reported elsewhere [38]. Admission arterial blood pH, representing one measure of metabolic acidosis, was added into the model. Adding pH removed the independent significance of lactate, creatinine, and platelets, so these were removed from the model; only haematocrit, parasite count, white cell count and SGOT remained significant factors associated with levels of Ang-2 (Model B, Table 5). This supported the hypothesis that Ang-2 was not directly correlated with lactate but both of them (together with renal dysfunction) were correlated with one common manifestation of severe disease - metabolic acidosis.

## Conclusions

Plasma concentrations of Ang-2 and the Ang-2/Ang-1 ratio were confirmed as independent predictors of death in Vietnamese adults with severe falciparum malaria, as seen in all other adequately powered severe malaria series studied to date. These include series of adult severe malaria from Indonesia [24], adult CM from India [25], and paediatric CM series from Uganda [26,27] and Malawi [28,29]. Patterns of immunostaining of Ang-1, Ang-2 and Tie-2 in malaria cases were altered in fatal severe malaria compared to fatal non-malaria cases, especially in neurons and astroglial cells. However, there were no specific patterns of Ang-1, Ang-2 and Tie-2 expression, either of neuronal, astroglial or vascular, that differentiated CM from non-cerebral severe malaria deaths. Activation of the Ang-Tie-2 pathway in severe malaria was related to acidosis, the number of severity criteria and outcome, but was not a specific event in the brain during CM.

Previous pathological studies of CM have demonstrated the presence of EC activation in the cerebral microvasculature [9], which occurs as part of a wider systemic endothelial activation in severe malaria, and which is not specific to cases that develop coma [10]. The pathophysiology of endothelial activation in CM has been hypothesized to occur in response to both systemic factors (such as the release of cytokines or a soluble malaria toxin) and local vasoactive substances such as VEGF or WPB products such as Ang-2. The expression of mediators, which could influence endothelial reactivity in the brain in CM has been previously examined, including inducible nitric oxide synthase [41], the hypoxic inducible protein HIF-1a and activation of the VEGF signalling pathway [42]. Therefore this methodology was extended to examine the expression of the angiopoietins and their receptor Tie-2 in the brain in CM and NCM cases, to determine the extent to which they were specific to CM and whether this pathway showed a direct link to the clinical features of coma before death.

The lack of significant differences in the patterns or extent of staining of any of these constituents between CM and NCM cases is consistent with recent data using systemic measures of activation of the Ang-2-Tie2 pathway, where elevated plasma levels of Ang-2 were no higher in CM than in NCM [24]. Taken together, these results do not support elevated Ang-2 as a predictive biomarker for cerebral malaria, as previously proposed [25,26]. Indeed in the current study, circulating Ang-2 was elevated less in those with CM as the only manifestation than in those with other severe malaria criteria.

The low levels of Ang-2 expression in brain ECs in fatal CM and NCM, despite elevated plasma concentrations of Ang-2, was unexpected. It is possible that exocytosis of cerebral endothelial WPBs at the time of death decreased immunoreactivity, due to prolonged EC activation, as stored Ang-2 is rapidly released from WPBs on activation [18]. Alternatively, raised Ang-2 levels in serum may reflect release from the extra-cerebral systemic circulation in both CM and NCM.

As well as its independent association with fatal outcome, Ang-2 was associated with number of organ complications, metabolic acidosis, and acute renal failure, as previously shown [24]. In contrast, the other major WPB product increased in severe malaria, vWF, is not associated with either lactate/metabolic acidosis or fatal outcome in severe malaria [39,43]. Taken together, these findings do still suggest a specific pathogenic role for Ang-2 in the pathways leading to death in severe malaria. Ang-2 competitively binds to Tie-2 receptors blocking the homeostatic effects of Ang-1, increasing endothelial activation and sensitising ECs to further activation and injury [19]. Ang-2 related amplification of endothelial activation, injury and microvascular sequestration may thus play a key role in impairment of microvascular perfusion in severe malaria.

Potential limitations to interpretation of immunohistochemical staining patterns include post mortem artefact, which may alter staining due to diffusion or degradation of proteins, and necessitated the use of control cases. Each death in an autopsy series gives a ‘snapshot’ effect of pathology in an individual case with varying treatment, time to death and clinical complications of disease, which differ between cases. Inferring a single unifying pattern of pathology with temporal sequence from a group of such cases may therefore be difficult. Severe malaria in Vietnamese adults is a multi-organ process with high prevalence of renal, liver, respiratory and metabolic dysfunction as well as anaemia and cerebral malaria. Hence several factors may affect the expression of a particular marker as judged by immunohistochemistry. Despite this, it was reassuring that the data on plasma levels of Ang-1, Ang-2 and Tie-2 were in keeping with previously published series on these mediators in severe and fatal malaria [24-29,39]. This lends weight to the findings that despite the significant increases in Ang-2 and Ang2/Ang-1 ratio in severe malaria, there was no evidence that this was a process that was specifically upregulated in the brain in CM *versus* NCM cases.

The analysis of the prognostic value of Ang-2 in severe malaria confirmed, as found in previous studies, that this marker is predictive of fatal outcome, at a level superior to previously studied prognostic biomarkers such as serum lactate. However most studies use a single (admission) value of serum Ang-2 and have not studied the kinetics during severe disease. Lactate levels fall rapidly after treatment but in contrast remain high or rise in patients with fatal outcome despite treatment [44,45]. Data on the kinetic responses of Ang-2 and Ang-2/Ang-1 ratio following treatment would be valuable in interpreting the specificity of Ang-2 increases in severe malaria.

The effects of Ang-1, Ang-2 and Tie-2 signalling on neurons and glial cells have not been as well studied as endothelium. It was notable that expression of Ang-1, Ang-2 and Tie-2 differed in neurons and glial cells in fatal malaria cases compared to controls. Recent studies indicate that Tie-2 signal transduction might have neuroprotective and mitogenic effects on neuronal cells. Ang-1-Tie-2 signalling promotes neural outgrowth from dorsal root ganglion cells [46], supports neuronal differentiation in neural progenitor cells [47], protects against neuronal apoptosis [48] and the effects of oxygen and glucose starvation [49], both of which may occur in the brain during the coma of CM. For these reasons, the increased neuronal Ang-1 expression in association with microvascular haemorrhages may reflect an adaptive, neuroprotective response to cerebral disease, where haemorrhages reflect focal damage to BBB function which are associated with perivascular edema formation [50]. In a murine model of angiogenesis during experimental autoimmune encephalomyelitis, temporal changes in neuronal and glial expression of Ang-1 and vascular expression of Tie-2 in the spinal cord was seen in concert with BBB permeability changes [51]. Ang-1 effects on neurons are not exclusively limited to Tie-2-receptor dependence, as Ang-1 can induce neurite outgrowth via the β1-integrin receptor on neurons [52]. Other studies have also found a positive effect of Ang-2 on neurogenesis and migration of neuroblasts [53]. It is therefore possible that increased expression of both Ang-1 and Ang-2 on neuronal cells are neuroprotective responses in severe malaria whether or not this involves coma.

Despite the finding that Ang-2 is associated with poor outcome, the immunohistochemical staining and ELISA data provided no evidence for direct involvement of this pathway in the genesis of coma by increases in CM *versus* NCM patients. The strong correlation between plasma Ang-2 levels and Ang2/Ang1 ratio with metabolic acidosis and increasing number of clinical severity criteria suggests an association with widespread endothelial activation in severe disease, even if this does not have a specific effect on BBB function in the genesis of coma. However the Ang-2-Tie-2 pathway could be studied using *in vitro* models of BBB function in the presence of sequestered, malaria-infected erythrocytes to investigate this further. Given the association of plasma Ang-2 with acute renal failure in this and other series, and the relationship with metabolic acidosis in the multivariate analysis, further studies should examine the role of the Ang-Tie-2 pathway in regulating blood flow and microvascular pathology in the kidney in adult severe malaria patients.

## Abbreviations

CM: Cerebral Malaria; NCM: Non-Cerebral Malaria; BBB: Blood–Brain Barrier; Ang-1: Angiopoietin-1; Ang-2: Angiopoietin-2; vWF: von Willebrand Factor; EC: Endothelial Cell; WPB: Weibel-Palade Body; GCS: Glasgow Coma Scale; ELISA: Enzyme-Linked Immunosorbent Assay; WHO: World Health Organization; CSF: Cerebrospinal Fluid; TNF: Tumor Necrosis Factor; SGOT: Serum Glutamic Oxaloacetic Transaminase; SGPT: Serum Glutamic Pyruvic Transaminase; BUN: Blood Urea Nitrogen.

## Competing interests

The authors declare that they have no competing interests.

## Authors’ contributions

PP carried the laboratory experiments, prepared and analysed the data obtained, drafted and finalized the manuscript for submission with contributions from authors. IM helped supervise and perform the laboratory experiments, analyse results and reviewed the manuscript. NTHM, NPJD, NHP, TTH, NJW, GDHT collected samples and data for the study. NJW, NPJD, TWY, NMA, GDHT conceived of the study, helped analyse results, wrote and reviewed the manuscript. All authors have read and approved the final manuscript.

## Supplementary Material

Additional file 1 Clinical details of malaria cases used in the immunohistochemical study. Click here for file

Additional file 2 Clinical details of control cases in the immunohistochemical study. Click here for file

Additional file 3 Angiogenic markers expression in different cellular component across brain areas (pooled malaria cases and non-malaria controls). Click here for file

Additional file 4 Angiogenic markers expression in different cellular component across brain areas (only malaria cases). Click here for file
